# Consumption of drugs for Alzheimer’s disease on the Brazilian private market

**DOI:** 10.11606/s1518-8787.2023057005128

**Published:** 2023-10-30

**Authors:** Evani Leite de Freitas, Sabrina Calil-Elias, Rafael Santos Erbisti, Branca Grinberg-Weller, Elaine Silva Miranda

**Affiliations:** I Universidade Federal Fluminense Faculdade de Farmácia Programa de Pós-graduação em Administração e Gestão da Assistência Farmacêutica Niterói RJ Brazil Universidade Federal Fluminense . Faculdade de Farmácia . Programa de Pós-graduação em Administração e Gestão da Assistência Farmacêutica . Niterói , RJ , Brazil; II Universidade Federal Fluminense Faculdade de Farmácia Departamento de Farmácia e Administração Farmacêutica Niterói RJ Brazil Universidade Federal Fluminense . Faculdade de Farmácia . Departamento de Farmácia e Administração Farmacêutica . Niterói , RJ , Brazil; III Universidade Federal Fluminense Instituto de Matemática e Estatística Departamento de Estatística Niterói RJ Brazil Universidade Federal Fluminense . Instituto de Matemática e Estatística . Departamento de Estatística . Niterói , RJ , Brazil; IV Universidade Federal Fluminense Faculdade de Farmácia Niterói RJ Brazil Universidade Federal Fluminense . Faculdade de Farmácia . Niterói , RJ , Brazil

**Keywords:** Alzheimer’s Disease, Drug Utilization, Dementia, Prescription Drugs, Drugs from the Specialized Component of Pharmaceutical Care

## Abstract

**OBJECTIVE:**

To analyze the consumption of drugs for Alzheimer’s disease on the Brazilian private market and its geographical distribution from 2014 to 2020.

**METHODS:**

National data from the Brazilian National System of Controlled Product Management were used, regarding sales of donepezil, galantamine, rivastigmine, and memantine from January 2014 to December 2020. Sales data were used as a proxy for drug consumption and expressed as defined daily dose/1,000 inhabitants/year at national, regional, federative unit and microregion levels.

**RESULTS:**

Drug consumption went from 5,000 defined daily doses/1,000 inhabitants, in 2014, to more than 16,000/1,000 inhabitants, in 2020, and all federative units showed positive variation. The Brazilian Northeast had the highest cumulative consumption in the period but displayed microregional disparities while the North region had the lowest consumption. Donepezil and memantine were the most consumed drugs, with the highest growth in consumption from 2014 to 2020.

**CONCLUSION:**

The consumption of medicines indicated to treat Alzheimer’s disease tripled in Brazil between 2014 and 2020, which may relate to the increase in the prevalence of the disease in the country, greater access to health services, and inappropriate use. This challenges managers and healthcare providers due to population aging and the increased prevalence of chronic-degenerative diseases.

## INTRODUCTION

Demographic transition is a population phenomenon in several countries ^1^ . The Brazilian population aged over 60 years represented 12.8% of all residents in 2012, increasing to 15.4% in 2018, representing over 30 million people. In 2018, this age group had the highest concentrations in the Brazilian Southeast (17.1%) and South (16.9%), but all major regions followed the trend of population aging ^2 , 3^ .

The increase in life expectancy is related to the growing prevalence of chronic-degenerative diseases such as dementia ^4^ , a neurological condition with symptoms such as the deterioration of cognitive and behavioral capacity and impaired memory and language use, which greatly affects patients’ quality of life ^5^ . The worldwide prevalence of dementia more than doubled from 1990 to 2016 and population aging configures a key factor in this growth ^6^ . Brazil stands out in this scenario with the second highest global estimate in relation to the age-standardized prevalence ^6^ . National studies still differ regarding the indicators of the disease in the country, hindering its reliable mapping ^7^ .

Alzheimer’s disease (AD) is the leading cause of dementia, associated with 60–70% of cases ^5^ . AD is a neurodegenerative disease associated with the accumulation of two pathogenic proteins, amyloid β peptide and tau protein, leading to neuronal dysfunction and loss and ultimately to the progression of cognitive impairment ^8^ .

This disease has no cure, and its pharmacological treatment is based on the use of acetylcholinesterase inhibitors (AChEi) and NMDA-type glutamate receptor antagonists to increase the supply of acetylcholine in synaptic clefts and reduce glutamatergic excitotoxicity, respectively ^9^ . The AChEi class includes donepezil, galantamine, and rivastigmine, and the latter class, only memantine. The Clinical Protocols and Therapeutic Guidelines (CPTG) for Alzheimer’s Disease ^9^ include all these drugs, establishing treatment guidelines for the disease, which is offered by the C *omponente Especializado de Assitência Farmacêutica* (CEAF – Specialized Component of Pharmaceutical Care). The acquisition on the National Health System (SUS) is conditioned to the delivery of specific documents and depends on their prior evaluation, which may exclude applicants ^10^ . Out-of-pocket acquisition can represent a source of access to medicines for these patients.

Ordinance No. 344, of May 12, 1998, regarding controlled substances, includes all indicated drugs to treat Alzheimer’s disease; thus, their trade is subject to record keeping. Private pharmacies and drugstores currently conduct this process, periodically and electronically sending data to the *Agência Nacional de Vigilância Sanitária* (Brazilian Health Regulatory Agency – ANVISA) ^11 , 12^ . All due information is sent to the *Sistema Nacional de Gerenciamento de Produtos Controlados* (SNGPC – Brazilian National System of Management of Controlled Product) and these data are publicly accessible, according to the 2016 Brazilian Open Data Policy ^13^ .

This study aims to analyze the consumption profile of Alzheimer’s drugs in Brazil using a publicly accessible database to evaluate trends in the consumption of these drugs in the private market.

## METHODS

### Study Design and Data Collection

This is a descriptive cross-sectional study. SNGPC dispensing records, made available by ANVISA (at the Brazilian Open Data Portal, in the section Sale of Controlled Drugs and Antimicrobials – Industrialized Drugs ^14^ ) were used as a source of data on drug consumption. The used data came from spreadsheets downloaded from May to September 2021 in an CSV format, using Excel ^®^ (2016) and its integrated tool PowerQuery, which offers ETL functions (extract, transform, and load data) to process and then analyze broad databases ^15^ .

The medications included in this study were all those marketed from January 2014 to December 2020 that included the authorized active ingredients to treat AD in Brazil, namely: rivastigmine, donepezil, galantamine, and memantine, in all available presentations, including combinations. The 10mg/g memantine presentation was excluded from this analysis since it was impossible to find its package leaflet to confirm dose per pharmaceutical unit. In an exploratory analysis of the data, we observed that the percentage of sales of this presentation totaled less than 0.1% of all other annuals sales. Thus, this presentation was excluded as we deemed it a small loss for this study.

### Statistical Analyses

For consumption analysis, defined daily doses (DDD) were used, a unit of measurement employed to evaluate trends in the use of medicines and to compare population groups as it remains stable despite price fluctuations, packaging changes, or presentation ^16^ . To find the DDD of each drug, its ATC (anatomical therapeutic chemical) classifications and corresponding DDD were searched ^17^ (Chart). Donepezil and memantine were analyzed separately due to the absence of a designated DDD for the commercialized association. We decided to clear this point with the Collaborating Centre of the World Health Organization (WHO), responsible for coordinating the ATC/DDD system. Based on this communication, the described form of analysis was chosen.

**Chart:** Antidementia drugs, ATC codes, and defined daily doses according to their international classification ^17^ .

Pharmaceuticals	Donepezil	Galantamine	Rivastigmine	Rivastigmine	Memantine
ATC Code	N06DA02	N06DA04	N06DA03	N06DA03	N06DX01
Administration route	Oral	Oral	Oral	Transdermal	Oral
DDD	7.5 mg	16 mg	9 mg	9.5 mg	20 mg

ATC: anatomical therapeutic chemical; DDD: defined daily dose.

Consumed amounts were estimated according to the DDD indicator per 1,000 inhabitants for each analyzed year, providing a population estimate of the use of these drugs. For this calculation, the population projections made available annually by the *Instituto Brasileiro de Geografia e Estatística* (Brazilian Institute of Geography and Statistics – IBGE) were considered, with a reference date of July 1 for each year ^18^ .

The formulas to calculate the indicators are shown below ^19^ .


DDD=( number of acquire packages )×( number of DDD per package )

$$DDD/1,000\text{ inhabitants/year }=\frac{(\text{ Use in DDD/year) }}{\left(\text{ number of inhabitants }\right)}\times 1,000$$

Annual consumption data for each active ingredient were obtained at the municipal level, identifying the respective unit of the federation or Federal District. For each evaluated year, information was aggregated into 558 microregions based on the compatibility of spatial scales. The territorial meshes relating municipalities and microregions were obtained according to the IBGE classification ^20^ .

Consumption classes to analyze spatial distributions were defined by calculating the quartiles of the indicator, expressed in DDD/1,000 inhabitants (25, 50, and 75%) considering the entire period of analysis (2014 to 2020). Thus, four consumption ranges were specified (in DDD/1,000 inhabitants/year): 0 to 103; 103 to 240; 240 to 466; and 466 to 491,376.

R software ^21^ (2021) was used to analyze the quantitative data and elaborate graphs and maps to determine consumption by period and place.

## RESULTS

Aggregate analysis ( Table ) of all drugs included in the study shows that national consumption went from 5,000 DDD/1,000 inhabitants in 2014 to more than 16,000 DDD/1,000 inhabitants in 2020, an increase of more than 200%. Moreover, consumption in all federal units varied positively.

**Table:** Aggregate and cumulative consumption of antidementia drugs sold from 2014 to 2020 by state and macroregion according to DDD/1,000 inhabitants. Brazil, 2022.

Region and Federation Unit	2014	2015	2016	2017	2018	2019	2020	Cumulative	Variation 2014–2020 (%)
North								5,831	
Rondônia	76	99	121	128	191	262	310	1,186	307
Acre	51	71	87	91	102	120	123	646	142
Amazonas	59	85	109	126	128	129	141	777	138
Roraima	49	70	75	91	93	111	121	612	145
Pará	92	120	150	143	161	189	209	1,064	126
Amapá	26	40	56	72	76	91	109	471	316
Tocantins	74	103	126	160	181	203	227	1,075	207
Northeast								22,561	
Maranhão	60	85	108	134	161	188	201	939	237
Piauí	109	148	191	231	285	325	338	1,628	210
Ceará	183	248	302	346	385	446	475	2,386	159
Rio Grande do Norte	191	259	329	396	499	592	2,374	4,639	1,145
Paraíba	276	328	366	422	478	533	596	2,998	116
Pernambuco	210	236	262	299	360	416	1,443	3,225	587
Alagoas	72	106	144	186	244	293	290	1,336	300
Sergipe	98	116	150	190	234	297	336	1,421	245
Bahia	93	122	155	395	2,633	281	312	3,990	236
Southeast								17,613	
Minas Gerais	300	388	488	591	698	833	931	4,229	211
Espírito Santo	192	263	339	403	461	533	583	2,774	203
Rio de Janeiro	495	577	668	1,796	1,409	815	837	6,597	69
São Paulo	596	873	382	448	506	578	631	4,013	6
South								13,937	
Paraná	245	286	358	415	2,013	507	559	4,383	128
Santa Catarina	242	295	372	444	514	587	606	3,060	150
Rio Grande do Sul	440	468	559	1,115	742	854	2,315	6,493	426
Midwest								14,012	
Mato Grosso do Sul	203	255	306	379	410	489	553	2,596	173
Mato Grosso	99	134	165	192	222	290	336	1,438	239
Goiás	221	268	337	464	466	530	569	2,854	158
Federal District	253	318	4,344	442	538	599	630	7,124	149
Brazil	5,005	6,363	11,048	10,100	14,192	11,091	16,155		223

DDD: defined daily dose.

In 2014, the states of São Paulo (596 DDD/1,000 inhabitants), Rio de Janeiro (494 DDD/1,000 inhabitants), and Rio Grande do Sul (440 DDD/1,000 inhabitants) showed the highest consumption of AD medication, whereas Rio Grande do Sul (2,314 DDD/1,000 inhabitants), Rio Grande do Norte (2,373 DDD/1,000 inhabitants), and Pernambuco (1,442 DDD/1,000 inhabitants) did so in 2020. Rio Grande do Norte and Pernambuco showed significant variation in consumption in relation to the beginning of the historical series (1,144.49 and 586.67%, respectively). These federative units showed the highest consumption of drugs for AD in 2020.

The Northeast showed the highest cumulative consumption over the seven evaluated years (22,561 DDD/1,000 inhabitants) and the greatest variation in consumption between 2014 and 2020. The Brazilian Southeast features in second place, with 17,612 DDD/1,000 inhabitants from January 2014 to December 2020. The Brazilian North region had the lowest cumulative consumption in the country (5,830 DDD/1,000 inhabitants).

**Figure 1:**
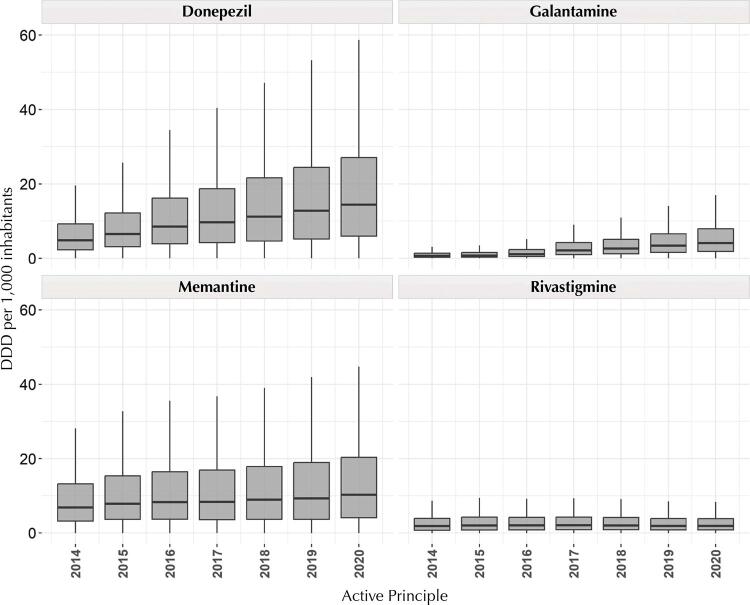
Difference in consumption of drugs to treat AD in Brazil from 2014 to 2020 in DDD/1,000 inhabitants. Brazil, 2022.

AD: Alzheimer’s disease; DDD: defined daily dose.

We found a higher magnitude of donepezil and memantine consumption than that of other drugs ( Figure 1 ). Moreover, using medians as reference, donepezil consumption increased about 150 DDD for every 1,000 inhabitants from 2014 to 2020 and that of memantine, almost 100 DDD/1,000 inhabitants; more expressive variations than those of galantamine and rivastigmine, comparatively. Moreover, our analysis of the interquartile ranges for donepezil and memantine show greater variability in each sales operation and more expressive sales volumes in our historical series ( Figure 1 ).

**Figure 2:**
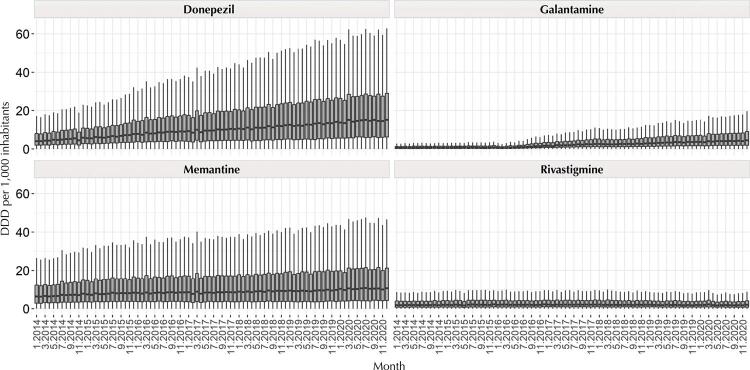
Monthly evaluation of the consumption of drugs to treat AD in Brazil, from 2014 to 2020 in DDD/1,000 inhabitants. Brazil, 2022.

AD: Alzheimer’s disease; DDD: defined daily dose.

Monthly analysis indicates that differences in the magnitude of consumption remained throughout the period, showing no seasonal or periodic trends ( Figure 2 ).

**Figure 3:**
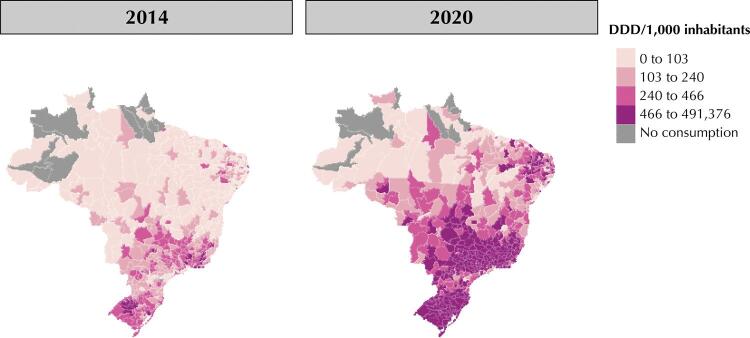
Consumption of drugs for AD treatment in Brazil, from 2014 to 2020, in DDD/1,000 inhabitants and according to microregions. Brazil, 2022.

AD: Alzheimer’s disease; DDD: defined daily dose.

Regarding spatial distribution, we analyzed the consumption of drugs in the Brazilian micro-regions. In 2014, it totaled 103 DDD/1,000 inhabitants in most of the country, with micro-regions without sales records, concentrated especially in the North region. In that year, the Southeast and South concentrated most of the consumption of the analyzed drugs as they contained all the microregions that consumed more than 466 DDD/1,000 inhabitants/year ( Figure 3 ). We found 12 Southeastern microregions in this upper consumption range, with the largest record in the state of São Paulo (25,070 DDD/1,000 inhabitants/year in the Limeira microregion), and the others distributed across Minas Gerais and Rio de Janeiro—with five and four microregions in this consumption range, respectively, in 2014. In the same year, the South had seven microregions in the state of Rio Grande do Sul consuming from 471 to 813 DDD/1,000 inhabitants/year.

Throughout the historical series, we can see that the magnitude of consumption rises in Brazil, especially on the South and Southeast, which showed a higher proportion of records in the upper range, above 466 DDD/1,000 inhabitants/year ( Figure 3 ).

The Northeast showed the highest accumulated consumption of drugs in the national scenario, with micro-regional disparities: In 2020, the states of Rio Grande do Norte (10), Paraíba (8), and Pernambuco (6) concentrated the microregions that consumed more than 466 DDD/1,000 inhabitants, totaling only 37 (approximately 20%) among the 187 Northeastern microregions. Consumption decreases outside these areas and toward the local inland ( Figure 3 ).

Regarding the Brazilian North, only one microregion in Rondônia and another in Pará, consumed more than 466 DDD/1,000 inhabitants in 2020. The highlight of this region stems from the maintenance of areas with consumption voids, especially in the states of Amazonas and Pará ( Figure 3 ).

## DISCUSSION

The consumption of drugs to treat AD increased by more than 200% from 2014 to 2020, representing an average growth rate of 21.56% per year. All federative units and major regions showed positive consumption variations. Similarly, pharmaceutical market data from other countries shows the growing demand for such drugs. Recent analysis of the international pharmaceutical market showed a 55.74% increase in the consumption of these drugs from 2008 to 2018, driven mainly by low- and middle-income countries such as Brazil ^22^ .

Results of the 2016 Global Burden of Disease Study (GDB 2016) indicate a global growth of 117% in dementia cases (or 26.6 million people) from 1990 to 2016 ^6^ . In Brazil, estimates suggest that, in 1990, 472,667 people had dementia (317.58/100,000 inhabitants), rising to 1,702,402 (785.73/100,000 inhabitants) in 2019 ^23^ .

We face the expectation of the worldwide growth in the prevalence of AD and other dementias. Projections for Brazil indicate that it will affect, on average, 5,666,116 people in 2050, an increase above 200% compared to 2019 ^24^ . In line with these estimates, the demographic profile indicates a rapid growth of the population aged 65 years or older (which represented 7.6% of its population in 2010), reaching 38% of the total population in 2050 ^25 , 26^ . Thus, the consumption profile we observed would tend to increase.

The consumption increase we found can also relate to aspects such as greater access to health services and/or some level of inadequate use of the analyzed drugs. The absence of national data on the prevalence of dementia in Brazil is notable. Thus, this study suggests a possible increase in this prevalence that deserves investigation.

A 2017 study showed improvements in the supply of health services in Brazil from 2000 to 2016, with greater access to primary care and services of medium and high complexity, including the provision of private supplementary services ^27^ . This improvement may be related to greater access to diagnostic services and the indication of treatment. Moreover, the expected trend of increasing prevalence of AD has already been reported for lower-middle-income countries ^28^ .

Diagnosis can offer challenges. Factors such as the symptomatic similarity between AD and other dementias and syndromes related to cognitive deterioration, can lead some patients, suffering from the latter, to be misdiagnosed and treated for AD ^29^ . Diagnostic accuracy is important to prevent potential risks derived from medication use. Correctly assessing a picture of cognitive deterioration can optimize pharmacological therapy and reduce the occurrence of potential adverse events ^29 , 30^ .

Regarding the analysis of national drug consumption, donepezil and memantine were more consumed than galantamine and rivastigmine since the beginning of the evaluated historical series, data corroborated in the literature ^22^ . Acetylcholinesterase inhibitors (AChEi) are the first line of treatment for mild to moderate AD, and donepezil is the only such drug indicated for all stages of the disease ^9^ . The recommendation (or indication) of donepezil for all stages of AD may be an important factor contributing to the greater magnitude of its consumption. Moreover, it is the oldest drug on the market for AD ^31^ , which may be associated with more consolidated prescribing habits.

Regarding the analysis of microregional distribution, Southern and Southeastern microregions predominate in the range of higher consumption, showing a more uniform distribution throughout the territory. The Brazilian South and Southeast show the highest proportion of older adults in the country ^3^ , which may contribute to this observation. The Brazilian Northeast showed a different situation. It had the highest cumulative consumption of our historical series but an irregular distribution, suggesting greater acquisition disparities.

Access to health services can be understood under different aspects, including purchasing capacity and geographical accessibility. Purchasing capacity addresses the adequacy between the cost of using health services and the ability to pay individuals, whereas geographical accessibility refers to the distance between the desired service and the user and the means of transport and travel time ^32 , 33^ .

A 2006 study showed that these two factors strongly influence access to health services in Brazil ^27^ . According to the authors, residents of the South and Southeast had greater access to health services, despite marked social inequalities in the South. Moreover, the improvement of access over a five-year period was greater in these more socioeconomically developed regions ^27^ . More recently, a study on regional health inequalities evaluated the internalization of the development and supply of services in the Brazilian South and Southeast, whereas the Northeast, despite its socioeconomic development, shows a high concentration in a few areas of greater economic activity ^34^ , an aspect that may be related to our results.

Other studies corroborate the relevance of purchasing capacity for the effective access to drugs both in private and public markets and access inequalities ^35 , 36 , 37^ . Moreover, the acquisition of drugs in the private market may impair family incomes ^38^ or patients may be unable to acquire drugs ^35 , 36^ , compromising therapeutic results ^39^ .

Acquisition in the private market may occur due to the absence of a drug in the public system ^32^ . It was only on the 2017 edition of the CPTG that memantine and rivastigmine transdermal patches were added to the national guideline. Currently, all approved drugs (but not all presentations) to treat AD feature in the 2022 National Relation of Essential Medicines ^40^ and are dispensed by CEAF according to guidelines established by the CPTG ^9^ .

Despite the incorporation into the national health system at the same time, disparities remain in the administrative process necessary to effect public procurement. The added rivastigmine presentations—transdermal patch 9 and 18mg—were coded in the *Sistema de Gerenciamento da Tabela de Procedimento, Medicamentos e OPM* (SIGTAP – Table of Procedures, Medicines, and OPM Management System) in 2017 ^41^ , whereas it only occurred for memantine 10mg in August 2019 ^42^ . This succession of facts may have influenced the public procurement of these drugs and the maintenance of low consumption of rivastigmine in the private market. On the other hand, a possible reduction in the consumption of memantine may have been absent in our observations due to our period of analysis.

This study has limitations. First, it used sales data as a proxy for use and does not take into consideration any events occurred after dispensation. Thus, data indirectly portray patients’ use ^43^ . This study also limited itself to the private market, excluding information about public market acquisition or dispensation in this sector.

We should also highlight the possibility of corrections to the SNGPC records over time by the establishments that use it; therefore, those aiming to perform this type of analysis should take into account possible modifications. Moreover, there is uncertainty regarding the use of rivastigmine, also approved to treat dementia associated with Parkinson’s disease, although excluded from the CPTG of this condition.

Despite the limitations, drug sales data can provide important information on drug consumption. We obtained data from a database open to the public and its employment corroborates the importance of public transparency regarding the use of secondary open databases for research.

This study brings unprecedented results by analyzing national data on the consumption of medicines for AD based on a national and public-access database, expanding the knowledge about the disease in Brazil. Results may usefully guide the management of pharmaceutical services in Brazil and other public health policies to strategically cope with AD in the country.

## CONCLUSION

The consumption of medicines indicated for the treatment of AD tripled on the Brazilian private market from 2014 to 2020. This increase occurred in varying proportions in all federative units. The Brazilian Northeast showed the highest consumption and the largest increase in consumption in the period. However, we observed that this region displayed microregional disparities more often than the South and Southeast, indicating possible inequalities in access to health and medicines. Results corroborate the growing epidemiological importance of AD in Brazil and highlight the relevance of preparing its health structure for the increase in the prevalence of this disease and the demand for treatment. Due to its epidemiological importance, more studies on the disease must be conducted in Brazil, fostering public policies, and improving the management of care for AD patients.
