# Supplementary material for: Consumption of drugs for Alzheimer’s disease on the Brazilian private market
Source: Rev Saude Publica. 2023 Oct 30;57:83. doi: 10.11606/s1518-8787.2023057005128 (PMC10631752; doi:10.11606/s1518-8787.2023057005128)
Supplement: Translation

# Translation: Consumo de medicamentos para doença de Alzheimer no mercado privado brasileiro

**Authors:** Evani Leite de Freitas, Sabrina Calil-Elias, Rafael Santos Erbisti, Branca Grinberg-Weller, Elaine Silva Miranda

**Affiliations:** I Brazil Universidade Federal Fluminense. Faculdade de Farmácia. Programa de Pós-graduação em Administração e Gestão da Assistência Farmacêutica. Niterói, RJ, Brazil; II Brazil Universidade Federal Fluminense. Faculdade de Farmácia. Departamento de Farmácia e Administração Farmacêutica. Niterói, RJ, Brazil; III Brazil Universidade Federal Fluminense. Instituto de Matemática e Estatística. Departamento de Estatística. Niterói, RJ, Brazil; IV Brazil Universidade Federal Fluminense. Faculdade de Farmácia. Niterói, RJ, Brazil

**Keywords:** Doença de Alzheimer, Uso de Medicamentos, Demência, Medicamentos sob Prescrição, Medicamentos do Componente Especializado da Assistência Farmacêutica

## Abstract

**OBJETIVO:**

Analisar o consumo de medicamentos para a doença de Alzheimer no mercado privado brasileiro e sua distribuição geográfica entre os anos de 2014 e 2020.

**MÉTODOS:**

Foram utilizados dados do Sistema Nacional de Gerenciamento de Produtos Controlados relativos às vendas de donepezila, galantamina, rivastigmina e memantina, entre janeiro de 2014 a dezembro de 2020, em todo o território nacional. Os dados de venda foram utilizados como *proxy* para o consumo dos medicamentos, avaliado em dose diária definida (DDD)/1.000 habitantes/ano em nível nacional, regional, por unidade federativa e microrregião.

**RESULTADOS:**

O consumo dos medicamentos passou de 5.000 DDD/1.000 habitantes em 2014 para mais de 16.000 DDD/1.000 habitantes em 2020, e todas as unidades de federação apresentaram variação positiva. A região Nordeste apresentou o maior consumo acumulado no período, porém exibiu disparidades microrregionais. A região Norte apresentou o menor consumo. Os medicamentos mais consumidos foram donepezila e memantina, os quais também apresentaram maior crescimento do consumo no intervalo de tempo entre os anos de 2014 e 2020.

**CONCLUSÃO:**

O consumo de medicamentos para o tratamento da doença de Alzheimer triplicou no Brasil entre os anos de 2014 e 2020, o que pode estar relacionado ao aumento da prevalência da doença no país e/ou maior acesso a serviços de saúde, assim como estar ligado, também, à utilização inapropriada destes medicamentos. Este é um desafio para gestores e profissionais de saúde num cenário de envelhecimento populacional e aumento da prevalência de doenças crônico-degenerativas.

## INTRODUÇÃO

O processo de transição demográfica é um fenômeno populacional que ocorre em diversos países ^1^ . No Brasil, a população acima de 60 anos representava 12,8% da população residente total em 2012, passando para 15,4% em 2018, estando mais de 30 milhões de pessoas nessa faixa etária. Esse estrato etário apresentou, em 2018, as maiores concentrações no Sudeste (17,1%) e no Sul (16,9%), mas todas as grandes regiões do país seguem a tendência de envelhecimento populacional ^2 , 3^ .

O aumento da expectativa de vida relaciona-se com o crescimento da prevalência de doenças crônico-degenerativas, como as demências ^4^ , que são condições neurológicas que se manifestam com sintomas como deterioração da capacidade cognitiva e comportamental, prejuízo de memória e do uso da linguagem, com grande impacto na qualidade de vida dos portadores ^5^ . A prevalência mundial das demências mais do que dobrou entre 1990 e 2016, e o envelhecimento da população é um fator-chave nesse crescimento ^6^ . O Brasil tem destaque nesse cenário, com a segunda maior estimativa mundial ao comparar a prevalência padronizada por idade ^6^ . Apesar disso, estudos nacionais ainda divergem em relação aos indicadores da presença da doença no país, dificultando o mapeamento da condição de forma fidedigna ^7^ .

A doença de Alzheimer (DA) é a principal causa de demência, e está associada a 60% a 70% dos casos ^5^ . A DA é uma doença neurodegenerativa associada ao acúmulo de duas proteínas patogênicas, peptídeo β-amiloide e proteína tau, o que leva à disfunção e perda neuronal, e em última análise, à progressão do quadro de comprometimento cognitivo ^8^ .

**Quadro:** Fármacos antidemência, códigos ATC e doses diárias definidas segundo classificação internacional ^17^ .

Fármacos	Donepezila	Galantamina	Rivastigmina	Rivastigmina	Memantina
Código ATC	N06DA02	N06DA04	N06DA03	N06DA03	N06DX01
Via Adm.	Oral	Oral	Oral	Transdérmico	Oral
DDD	7,5 mg	16 mg	9 mg	9,5 mg	20 mg

ATC: *Anatomical Therapeutic Chemical*; DDD: dose diária definida.

Esta doença não tem cura e seu tratamento farmacológico se baseia na utilização de inibidores da enzima acetilcolinesterase (iAChE) e antagonistas do receptor de glutamato do tipo NMDA, buscando, respectivamente, aumentar a oferta de acetilcolina nas fendas sinápticas e reduzir a excitotoxicidade glutamatérgica ^9^ . Na classe dos iAChE estão donepezila, galantamina e rivastigmina, e na segunda classe, apenas a memantina. Todos estes fármacos estão incluídos nos Protocolos Clínicos e Diretrizes Terapêuticas (PCDT) para a Doença de Alzheimer ^9^ , estabelecendo as linhas de cuidado para a doença, cujo tratamento é ofertado por meio do Componente Especializado da Assistência Farmacêutica (CEAF). Esta dispensação pelo SUS está condicionada à entrega de documentos específicos: sua liberação depende da avaliação prévia destes documentos, não sendo aprovada para todos os solicitantes ^10^ . Neste sentido, a aquisição no mercado privado pode representar uma fonte de acesso aos medicamentos para os pacientes recusados.

Todos os medicamentos indicados para o tratamento da DA estão incluídos na Portaria nº 344 de 12 de maio de 1998, desta forma, sua comercialização está sujeita à escrituração. Atualmente, este processo é realizado por farmácias e drogarias privadas, com o envio periódico dos dados à Agência Nacional de Vigilância Sanitária (Anvisa), por meio eletrônico ^11 , 12^ . Esta escrituração é realizada no Sistema Nacional de Gerenciamento de Produtos Controlados (SNGPC), cujos dados são de acesso público, conforme a Política de Dados Abertos do Governo Federal, instituída em 2016 ^13^ .

O objetivo deste trabalho foi analisar o perfil de consumo dos medicamentos para Alzheimer no Brasil, utilizando base de dados de acesso público, a fim de avaliar tendências desse consumo no mercado privado.

## MÉTODOS

### Delineamento do Estudo e Fontes de Dados

Trata-se de um estudo descritivo, de tipo ecológico. Como fonte de dados de consumo de medicamentos utilizaram-se os registros de dispensação do SNGPC disponibilizados pela Anvisa por meio do Portal Brasileiro de Dados Abertos, na seção Venda de Medicamentos Controlados e Antimicrobianos – Medicamentos Industrializados ^14^ . Os dados utilizados neste trabalho são oriundos de planilhas baixadas entre maio e setembro de 2021, no formato CSV., utilizando-se o software Excel® (2016) e sua ferramenta integrada PowerQuery, que possibilita a execução de funções ETL (extrair, transformar e carregar dados) para o processamento e posterior análise de bases amplas de dados ^15^ .

Os medicamentos analisados neste trabalho foram todos aqueles comercializados no período de janeiro de 2014 a dezembro de 2020, que incluam os princípios ativos autorizados para o tratamento da DA no país, a saber: rivastigmina, donepezila, galantamina e memantina, em todas as apresentações disponíveis, incluindo associações. Excetua-se desta análise a apresentação de memantina de 10mg/g, pois não foi possível encontrar a bula de tal apresentação para confirmar a dose por unidade farmacêutica. Em uma análise exploratória dos dados foi possível observar que o percentual de venda desta apresentação dentre as demais foi menor do que 0,1% anualmente. Sendo assim, considerou-se que a exclusão desta apresentação seria uma perda de pequena monta para o estudo.

### Análise Estatística

Para a análise do consumo, foram utilizadas as doses diárias definidas (DDD), unidade de medida usada para avaliar tendências na utilização de medicamentos e úteis para comparações entre grupos populacionais, por não variar devido a flutuações de preço, modificações de embalagem ou apresentação ^16^ . Para encontrar a DDD de cada medicamento, foram consultadas suas classificações ATC ( *Anatomical Therapeutic Chemical* ) e posteriormente a DDD correspondente ^17^ ( Quadro ). Neste trabalho, donepezila e memantina foram analisados separadamente, pois não há DDD designada para a associação comercializada. Optou-se por esclarecer este ponto junto ao Centro Colaborador da Organização Mundial da Saúde responsável pela coordenação do sistema ATC/DDD, e a partir desta comunicação optou-se pela forma de análise aqui apresentada.

A quantidade de medicamentos consumida foi calculada segundo o indicador DDD por 1.000 habitantes para cada ano analisado, fornecendo uma estimativa populacional do uso dos medicamentos. Para o cálculo, foram consideradas as projeções populacionais disponibilizadas anualmente pelo Instituto Brasileiro de Geografia e Estatística (IBGE), com data de referência em 1º de julho de cada ano ^18^ .

Abaixo seguem as fórmulas utilizadas para o cálculo dos indicadores ^19^ .


$$DDD=\left(n^{\circ }\text{ de embalagens adquiridas }\right)\times \left(n^{\circ }\text{ de }DDD\text{ por embalagem }\right)$$

$$D/1.000\text{ habitantes/ano }=\frac{\left(\text{ utillzaçao em DDD/ano }\right)}{\left(n^{\circ }\text{ de habitantes }\right)}\times 1.000$$

Os dados de consumo anual de cada princípio ativo foram obtidos a nível municipal, com a identificação da respectiva unidade da federação ou Distrito Federal. A cada ano avaliado, as informações foram agregadas em 558 microrregiões, a partir de uma compatibilização das escalas espaciais. As malhas territoriais que relacionam município e microrregião foram obtidas segundo classificação do IBGE ^20^ .

As classes de consumo, para a análise da distribuição espacial, foram definidas a partir do cálculo dos quartis do indicador expressado em DDD/1.000 habitantes (25%, 50% e 75%) considerando todo o período de análise (2014 a 2020). Desta maneira, foram especificadas quatro faixas de consumo (em DDD/1.000 habitantes/ano): 0 a 103; 103 a 240; 240 a 466; 466 a 491.376.

Utilizou-se, também, o software R ^21^ (2021) para a análise dos dados quantitativos e elaboração dos gráficos e dos mapas, a fim de determinar o consumo por período e local.

## RESULTADOS

A análise agregada ( Tabela ) de todos os medicamentos incluídos no estudo, mostra que o consumo nacional passou de 5.000 DDD/1.000 habitantes em 2014 para mais de 16.000 DDD/1.000 habitantes em 2020, o que significa aumento de mais de 200%; o consumo em todas as UF também variou positivamente.

**Tabela:** Consumo agregado e acumulado de medicamentos antidemência vendidos de 2014 a 2020 por UF e por macrorregião,em DDD/1.000 habitantes. Brasil, 2022.

Região e Unidade da Federação	2014	2015	2016	2017	2018	2019	2020	Acumulado	Variação 2014–2020 (%)
Região Norte								5.831	
Rondônia	76	99	121	128	191	262	310	1.186	307
Acre	51	71	87	91	102	120	123	646	142
Amazonas	59	85	109	126	128	129	141	777	138
Roraima	49	70	75	91	93	111	121	612	145
Pará	92	120	150	143	161	189	209	1.064	126
Amapá	26	40	56	72	76	91	109	471	316
Tocantins	74	103	126	160	181	203	227	1.075	207
Região Nordeste								22.561	
Maranhão	60	85	108	134	161	188	201	939	237
Piauí	109	148	191	231	285	325	338	1.628	210
Ceará	183	248	302	346	385	446	475	2.386	159
Rio Grande do Norte	191	259	329	396	499	592	2.374	4.639	1.145
Paraíba	276	328	366	422	478	533	596	2.998	116
Pernambuco	210	236	262	299	360	416	1.443	3.225	587
Alagoas	72	106	144	186	244	293	290	1.336	300
Sergipe	98	116	150	190	234	297	336	1.421	245
Bahia	93	122	155	395	2.633	281	312	3.990	236
Região Sudeste								17.613	
Minas Gerais	300	388	488	591	698	833	931	4.229	211
Espírito Santo	192	263	339	403	461	533	583	2.774	203
Rio de Janeiro	495	577	668	1.796	1.409	815	837	6.597	69
São Paulo	596	873	382	448	506	578	631	4.013	6
Região Sul								13.937	
Paraná	245	286	358	415	2.013	507	559	4.383	128
Santa Catarina	242	295	372	444	514	587	606	3.060	150
Rio Grande do Sul	440	468	559	1.115	742	854	2.315	6.493	426
Região Centro-oeste								14.012	
Mato Grosso do Sul	203	255	306	379	410	489	553	2.596	173
Mato Grosso	99	134	165	192	222	290	336	1.438	239
Goiás	221	268	337	464	466	530	569	2.854	158
Distrito Federal	253	318	4.344	442	538	599	630	7.124	149
Brasil	5.005	6.363	11.048	10.100	14.192	11.091	16.155		223

UF: unidade de federação; DDD: dose diária definida.

Em 2014, São Paulo (596 DDD/1.000 habitantes), Rio de Janeiro (494 DDD/1.000 habitantes) e Rio Grande do Sul (440 DDD/1.000 habitantes) foram os estados com maior consumo dos medicamentos. Em 2020, o Rio Grande do Sul seguiu em destaque (2.314 DDD/1.000 habitantes), junto ao Rio Grande do Norte (2.373 DDD/1.000 habitantes) e Pernambuco (1.442 DDD/1.000 habitantes). Ambos estados nordestinos apresentaram variação expressiva do consumo em relação ao início da série histórica – 1.144,49% e 586,67%, respectivamente – e foram as unidades federativas de maior consumo de medicamentos para DA em 2020.

A região Nordeste apresentou o maior consumo acumulado ao longo dos sete anos avaliados, com 22.561 DDD/1.000 habitantes dispensadas no período. A região também foi a que apresentou maior variação no consumo entre 2014 e 2020. Em segundo lugar, aparece a região Sudeste, com 17.612 DDD/1.000 habitantes consumidas entre janeiro de 2014 e dezembro de 2020. A região Norte tem o consumo acumulado mais baixo do país (5.830 DDD/1.000 habitantes).

Pôde-se verificar que o consumo de donepezila e memantina tem maior magnitude em comparação aos demais fármacos ( Figura 1 ). Ademais, tomando como referência a mediana das observações, o consumo de donepezila aumentou aproximadamente 150 DDD para cada 1.000 habitantes entre 2014 e 2020, e a memantina, quase 100 DDD/1.000 habitantes, variações mais expressivas do que aquelas observadas para galantamina e rivastigmina, comparativamente. Ademais, a partir da análise dos intervalos interquartílicos para donepezila e memantina, com maior variabilidade dos quantitativos registrados em cada operação de venda, denotam-se volumes mais expressivos de venda ao longo da série histórica ( Figura 1 ).

**Figura 1:** Diferença de consumo dos fármacos utilizados no tratamento da DA no Brasil, de 2014 a 2020, em DDD/1.000 habitantes. Brasil, 2022.

DA: doença de Alzheimer; DDD: dose diária definida.

A análise mensal ao longo da série histórica indica que a diferença da magnitude do consumo se mantém ao longo de todo o período analisado, não indicando tendências relacionadas à sazonalidade ou períodos específicos ( Figura 2 ).

**Figura 2:** Evolução mensal do consumo dos fármacos utilizados no tratamento da DA no Brasil, de 2014 a 2020, em DDD/1.000 habitantes. Brasil, 2022.

DA: doença de Alzheimer; DDD: dose diária definida.

Em relação à distribuição espacial, analisou-se o consumo dos medicamentos nas microrregiões brasileiras. Em 2014, este consumo era de até 103 DDD/1.000 habitantes na maior parte do país, havendo microrregiões sem registro de vendas, concentradas majoritariamente na região Norte. Naquele ano, o consumo dos medicamentos analisados concentrou-se nas regiões Sudeste e Sul, onde localizaram-se todas as microrregiões que consumiram mais de 466 DDD/1.000 habitantes/ano ( Figura 3 ). No Sudeste, foram identificadas 12 microrregiões nesta faixa superior de consumo, sendo o maior registro no estado de São Paulo (25.070 DDD/1.000 habitantes/ano na microrregião de Limeira), e as demais distribuídas entre os estados de Minas Gerais e Rio de Janeiro, com 5 e 4 microrregiões nessa faixa de consumo, respectivamente, no ano de 2014. Na região Sul, neste mesmo ano, houve registro de sete microrregiões, todas localizadas no estado do Rio Grande do Sul, com consumos que variaram entre 471 e 813 DDD/1.000 habitantes/ano.

**Figura 3:** Consumo de medicamentos para o tratamento da doença de Alzheimer no Brasil, em 2014 e 2020, em DDD/1.000 habitantes, por microrregiões. Brasil, 2022.

DDD: dose diária definida.

Ao longo da série histórica, pode-se perceber que a magnitude do consumo se expande no país, com o destaque para as regiões Sul e Sudeste, onde há maior proporção de registros na faixa superior, acima de 466 DDD/1.000 habitantes/ano ( Figura 3 ).

A região Nordeste, onde ocorreu o maior consumo acumulado de medicamentos no cenário nacional, exibe disparidades microrregionais: Em 2020, as microrregiões que consumiram mais de 466 DDD/1.000 habitantes se concentraram nos estados de Rio Grande do Norte (10), Paraíba (8) e Pernambuco (6), e totalizam somente 37 (aproximadamente 20%) dentre as 187 microrregiões nordestinas. Fora destas áreas, e em direção ao interior da região, o consumo se reduz ( Figura 3 ).

Em relação ao Norte do país, em 2020 encontra-se apenas uma microrregião com consumo superior a 466 DDD/1.000 habitantes, em Rondônia, e outra localizada no Pará. O destaque da região fica por conta da manutenção de áreas com vazios de consumo, localizados primariamente nos estados do Amazonas e Pará ( Figura 3 ).

## DISCUSSÃO

O consumo dos medicamentos utilizados no tratamento da DA aumentou mais de 200% entre 2014 e 2020, representando uma taxa média de crescimento de 21,56% ao ano. Todas as unidades federativas e Grandes Regiões apresentaram variação positiva no consumo. Similarmente, dados do mercado farmacêutico de outros países mostram a crescente demanda por tais medicamentos. Análise recente do mercado farmacêutico internacional mostrou um aumento de 55,74% no consumo destes medicamentos entre 2008 e 2018, impulsionado principalmente por países de baixa e média renda, como o Brasil ^22^ .

Resultados do Estudo de Carga Global de Doença de 2016 (GDB 2016) indicam crescimento de 117% nos casos de demência em nível global - ou 26,6 milhões de pessoas, comparando-se os anos de 1990 e 2016 ^6^ . No Brasil, estima-se que em 1990 havia 472.667 pessoas com demência (317,58/100.000 habitantes), passando para 1.702.402 (785,73/100.000 habitantes) em 2019 ^23^ .

A perspectiva é de crescimento mundial na prevalência da DA e outras demências. Projeções para o Brasil apontam que em 2050 haverá, em média, 5.666.116 pessoas afetadas pela doença, mais de 200% de aumento em relação a 2019 ^24^ . Em linha com essas estimativas, o perfil demográfico indica um rápido crescimento da população com 65 anos ou mais, que representava 7,6% em 2010 e chegará a 38% do total de habitantes em 2050 ^25 , 26^ . Neste sentido, o perfil de consumo observado no presente estudo tende a ser crescente.

O aumento do consumo, observado no presente estudo, pode também ser analisado tendo em vista aspectos como maior acesso a serviços de saúde, e/ou algum nível de utilização inadequada dos medicamentos analisados. Cabe destacar que não existem dados nacionais sobre a prevalência de demências no país, neste sentido, o presente estudo traz uma perspectiva de possível aumento da prevalência que merece ser investigada.

Um estudo de 2017 apontou melhorias na oferta de serviços de saúde no Brasil entre 2000 e 2016, com maior acesso a serviços de atenção primária, de média e alta complexidade, incluindo oferta de serviços na rede privada, de caráter suplementar ^27^ . Esta melhoria pode estar relacionada ao maior acesso a serviços diagnósticos e à indicação de tratamento. Somando-se a isso, a tendência esperada de aumento da prevalência da DA já reportada para países de renda média-baixa ^28^ .

Acerca do diagnóstico, este pode apresentar desafios. Devido a fatores como a apresentação sintomatológica da DA guardar semelhanças com outros quadros demenciais e síndromes relacionadas à deterioração cognitiva, uma parcela de pacientes é diagnosticada com DA e recebe o tratamento indicado para a doença, apesar de não a apresentar de fato ^29^ .

A importância da acurácia diagnóstica em relação ao uso dos medicamentos avaliados reside no fato de que essa utilização não é isenta de riscos, e avaliar corretamente um quadro de deterioração cognitiva pode otimizar a terapêutica farmacológica e reduzir a ocorrência de potenciais eventos adversos ^29 , 30^ .

Em relação à análise do consumo nacional por fármaco, donepezila e memantina têm consumo de maior magnitude frente a galantamina e rivastigmina desde o início da série histórica avaliada, dados corroborados na literatura ^22^ . Os inibidores da acetilcolinesterase (iAChE) compõem a primeira linha de tratamento para quadros leves a moderados de DA, sendo a donepezila o único fármaco desta classe indicado em todos os estágios da doença ^9^ . A recomendação (ou a indicação) da donepezila para todos os graus da DA pode ser um fator importante que contribui para a maior magnitude de seu consumo. Outro ponto a ser considerado é que este é o medicamento mais antigo no mercado, dentre os demais utilizados na DA ^31^ , o que pode estar associado a hábitos de prescrição mais consolidados.

Em relação à análise da distribuição microrregional, predominam na faixa de maior consumo microrregiões localizadas no Sul e Sudeste do país, onde percebe-se distribuição mais uniforme ao longo do território. Sul e Sudeste são as regiões com a maior proporção de idosos do país ^3^ , o que pode contribuir para esta observação. Na região Nordeste, observa-se uma situação distinta. Esta região apresentou o maior consumo acumulado da série histórica avaliada, porém a distribuição é irregular na região, sugerindo maiores disparidades em relação à aquisição.

O acesso a serviços de saúde pode ser entendido sob diferentes aspectos, entre eles capacidade aquisitiva e acessibilidade geográfica. A capacidade aquisitiva trata da adequação entre o custo da utilização dos serviços de saúde e a capacidade de pagamento dos indivíduos, enquanto a acessibilidade geográfica diz respeito à distância entre o serviço desejado e o usuário, assim como aos meios de transporte e o tempo de deslocamento ^32 , 33^ .

Um estudo publicado em 2006 mostrou que esses dois fatores influenciam fortemente o acesso a serviços de saúde no Brasil ^27^ . Segundo os autores, residentes das regiões Sul e Sudeste tiveram maior acesso a serviços sanitários, apesar de marcadas desigualdades sociais na região Sul. Some-se a isso a observação de que a melhoria do acesso observada em um período de cinco anos foi maior nestas regiões, mais socioeconomicamente desenvolvidas ^27^ . Mais recentemente, um trabalho acerca de desigualdades regionais na saúde avaliou a interiorização do desenvolvimento e da oferta de serviços no Sul e Sudeste, enquanto no Nordeste, apesar do desenvolvimento socioeconômico ocorrido, observa-se alta concentração em poucas áreas de maior atividade econômica ^34^ , aspecto que pode estar relacionado com os resultados deste trabalho.

Outros estudos corroboram a relevância da capacidade aquisitiva para a efetivação do acesso a medicamentos, tanto no mercado privado quanto no público e iniquidades neste acesso ^35 , 36 , 37^ . Ainda, a aquisição dos medicamentos na rede privada pode levar ao comprometimento da renda familiar ^38^ ou o paciente pode acabar por não adquirir o medicamento ^35 , 36^ , comprometendo resultados terapêuticos ^39^ .

A aquisição na rede privada pode ocorrer pela ausência do medicamento na rede pública ^32^ . Neste sentido, foi apenas na edição de 2017 do PCDT que ocorreu a incorporação da memantina e da rivastigmina em sua apresentação de adesivos transdérmicos. Atualmente, todos os medicamentos – ainda que não todas as apresentações – aprovados para o tratamento da DA estão listados na Relação Nacional de Medicamentos Essenciais 2022 ^40^ , e são dispensados pelo CEAF, segundo linhas de cuidado estabelecidas nos PCDT ^9^ .

Apesar da incorporação ao SUS no mesmo momento, houve disparidades no processo administrativo necessário para efetivar as compras públicas. As apresentações de rivastigmina incorporadas – adesivo transdérmico 9mg e 18mg – tiveram código atribuído no Sistema de Gerenciamento da Tabela de Procedimentos, Medicamentos e OPM do SUS (SIGTAP) ainda em 2017 ^41^ , enquanto o processo só ocorreu para a memantina 10mg em agosto de 2019 ^42^ . Esta sucessão de fatos pode haver influenciado nas compras públicas destes medicamentos e na manutenção do baixo consumo de rivastigmina no mercado privado e, por outro lado, uma possível redução do consumo de memantina pode não ter sido observada devido ao período de análise do presente trabalho.

Este trabalho possui limitações. Em primeiro lugar, tomaram-se os dados de venda como *proxy* para o uso, e não foram considerados quaisquer eventos ocorridos após a dispensação; neste sentido, não retratam diretamente o uso pelos pacientes ^43^ . Ademais, o estudo limita-se ao mercado privado, não incluindo informações acerca de aquisições relativas ao mercado público ou dados de dispensação nesse setor.

Outro ponto a ser salientado é a possibilidade de correções nos registros do SNGPC ao longo do tempo pelos estabelecimentos que o utilizam; isso deve ser considerado por quem pretende realizar este tipo de análise, levando em conta a possibilidade de modificação. Somando-se a essas questões, a limitação acerca da incerteza quanto ao uso da rivastigmina, também aprovada para uso na demência, associada à doença de Parkinson, ainda que não esteja incluída nos PCDT desta condição.

Apesar das limitações supracitadas, os dados de venda de medicamentos podem fornecer dados importantes sobre o consumo de medicamentos. Os dados utilizados neste trabalho foram obtidos de base de dados aberta ao público, e sua utilização corrobora a importância da transparência pública em relação ao uso de bases de dados secundários, abertas, para fins de pesquisa.

O presente estudo traz resultados inéditos, ao analisar dados nacionais sobre o consumo de medicamentos para a DA obtidos de base de dados de alcance nacional e acesso público, ampliando o conhecimento sobre a presença da doença no país. Os resultados apresentados podem ser úteis para orientar a gestão da assistência farmacêutica no país e demais políticas públicas de saúde, visando ao enfrentamento estratégico da DA no país.

## CONCLUSÃO

O consumo de medicamentos para o tratamento da DA triplicou no mercado privado brasileiro entre 2014 e 2020. O aumento ocorreu em todas as unidades federativas em proporções variadas. A região Nordeste acumulou o maior consumo ao longo dos 7 anos, e foi também a região com o maior aumento do consumo no período. Porém, observou-se que, nessa região, as disparidades microrregionais são mais frequentes do que nas regiões Sul e Sudeste, denotando possíveis iniquidades no acesso à saúde e medicamentos. Os resultados deste trabalho corroboram a crescente importância epidemiológica da DA no país, e salientam a relevância de preparar a estrutura de saúde para o aumento de prevalência desta doença e da demanda por tratamento. É essencial que, por sua importância epidemiológica, sejam realizados mais estudos acerca da doença no Brasil, fomentando políticas públicas e melhorias na gestão do cuidado ao portador da DA.
